# Psychometric and Perceptometric Comparisons of the Perspectives of Orthodontists, Oral and Maxillofacial Surgeons, and Laypeople of Different Ages and Sexes towards Beauty of Female Jaw Angles (Intergonial Widths and Gonial Heights) on Frontal and Three-Quarter Views

**DOI:** 10.1155/2022/2595662

**Published:** 2022-11-08

**Authors:** Mehrnaz Moradinejad, Atefe Rekabi, Alireza Hashemi Ashtiani, Nastaran Atashkar, Vahid Rakhshan

**Affiliations:** ^1^Department of Orthodontics, School of Dentistry, Ahvaz Jundishapur University of Medical Sciences, Ahvaz, Iran; ^2^Department of Prosthodontics, School of Dentistry, Ahvaz Jundishapur University of Medical Sciences, Ahvaz, Iran; ^3^Department of Anatomy, Dental School, Azad University of Medical Sciences, Tehran, Iran

## Abstract

**Objectives:**

The jaw angle plays an important role in facial beauty. Therefore, this study is aimed at comparatively determining the range of most attractive female intergonial widths and gonial heights on Perceptometric frontal-view and three-quarter-view images, from the perspective of orthodontists, oral maxillofacial (OMF) surgeons, and laypeople of different ages and sexes.

**Methods:**

This prospective multivariate Perceptometric study was performed on 4191 esthetic scores given by 127 individuals to 33 Perceptometric face images. Frontal view and three-quarter-view photographs of a normal young woman were modified by image editing software to create two Perceptometric sets, one for the 24 gradual changes of intergonial width on the frontal view, and the other for the 9 vertical changes of the jaw angle on the three-quarter view. An online questionnaire was designed including 24 frontal and 9 oblique view photographs. The questionnaires' internal consistencies were almost perfect. Enrolled were 127 raters, including 33 orthodontists, 32 OMF surgeons, and 62 laypeople. The esthetics of different images were compared across different professions, across different ages, and between the sexes using 2-way MANCOVA, ANCOVA, and Bonferroni; the zones of esthetic jaw angles and also the sensitivity of judges to Perceptometric anatomical changes were assessed using 2-way RM-ANCOVA and Bonferroni (*α* = 0.05, *α* = 0.0056, *α* = 0.0021, and *β* = 0.05).

**Results:**

Orthodontists and surgeons gave the highest attractiveness scores to intergonial: interzygomatic ratio of 72.53%, while the best ratio was 74.45% for the laypeople. The *range* of beautiful intergonial is as follows: interzygomatic ratio was 72.53% to 86.03%. OMF surgeons and orthodontists gave the highest score to a gonial height of 4.5 mm above the mouth corner, while the laypeople gave the highest score to the gonial height of 4.5 mm below the mouth corner. The range of beautiful gonial height was from 4.5 mm above the mouth corner to 9 mm below the mouth corner. The education of observers may affect their perception of beauty; orthodontists tended differ from laypeople, overall and also specifically in the case of the highly attractive frontal images concerning the intergonial width changes. However, no such differences were detected between surgeons with orthodontists or laypeople. Although age did not affect the overall esthetic scores, it did affect the sensitivity of the judges to the anatomic changes. So did expertise, i.e., the expertise of judges affected their sensitivity to anatomical changes; orthodontists showed steeper slopes of esthetic preference alterations to anatomical changes, while laypeople had the gentlest slope of preference changes. Judges' sex did not affect either their overall esthetic preferences or their sensitivity to anatomic changes.

**Conclusion:**

Narrower female jaw angles and jaw angles that are vertically close to the level of the mouth corner may be unanimously more desirable. Thus, treatments aiming at widening the jaw angle of a woman or lowering it should be discouraged, at least in Persians. Orthodontists, but not surgeons, are more sensitive than laypeople to anatomic changes of the jaw angle. The judges' age can affect this perceptive sensitivity, but their sex cannot.

## 1. Introduction

Beauty is gaining an ever-increasing importance in orthodontics and other fields of dentistry [1–6]. Facial attractiveness has significant positive social and biological consequences; attractive individuals are often treated better by others, are psychosocially more successful and comfortable, and usually have higher verbal skills and social communications [7–12]. Therefore, the chief incentive for orthodontic patients is shifting increasingly to the improvement of their look [5], marking esthetics as a major area in orthodontics [1–6, 12–14].

To obtain appropriate esthetics, one should first define beauty and facial harmony [2, 15]. Several definitions have been proposed for an attractive face over the years, and many measures have been defined for this purpose [2, 16–18]. Newer definitions for describing an attractive face involve mathematical analyses and calculations or geometric morphometric methods based on landmarks' coordinates and the related linear and angular measurements [5, 6, 17, 19, 20].

The knowledge of esthetic preferences and attributes is of clinical and scientific importance. Maxillofacial and plastic surgeons aiming to reconstruct the face or perform a cosmetic surgical procedure can create a more beautiful face, if they have comprehensive knowledge about facial attractiveness and the influential factors in this respect [21]. Esthetic preferences can be researched via a the Perceptometrics method, which allows computerized, controlled manipulation of facial features within a defined range to create controlled photogrammetric alterations in the face to examine the esthetic preference range and ideals of experts or laypersons [6, 22–25].

Esthetic preferences may be a function of various factors. For instance, the psychological and educational factors affecting the perception of the observers may be important; laypeople versus clinicians can determine facial esthetics in a different way; thus, the evaluation of factors contributing to beauty from the perspective of experts such as oral and maxillofacial (OMF) surgeons and orthodontists versus laypeople is necessary [6, 15, 26, 27]. Other properties of observers may matter as well. For example, age, gender, physiological status, personality, and lifestyle of the rater, and similarity of the face to be rated to the face of the rater might all affect the score of facial attractiveness given by the raters [7, 16]. Perception of beauty might vary between males and females, different age groups, cultures, and ethnic groups; the level of agreement between two raters is usually 0.3 to 0.5; this value ranges from 0.8 to 0.9 among larger groups [7–9, 17, 21, 28, 29].

Aside from the perspectives of the evaluators, the other set of crucial factors affecting beauty is obviously the anatomical properties of the person to be judged. Facial soft tissue and its elements are critical to the person's attractiveness, and hence to orthodontic or surgical diagnosis and treatment planning [5, 15, 20, 30, 31].

Despite the significance of beauty, the literature on the anatomical factors influencing facial beauty is scarce and mostly limited to controversial and mostly small studies on soft-tissue profile [5, 6, 11, 13–15, 20, 22–25, 32]. Nevertheless, it is the frontal view that is most relevant to the patient's esthetic preferences and not the profile view, because the patient usually looks at his or her frontal or three-quarter oblique views in the mirror or in photographs and not at their profile views [33, 34].

One of the potentially affecting anatomical factors that has not been assessed is the gonial or jaw angle [35]. The gonial angle is formed between the mandible's inferior border and the ramus's posterior border. The gonial angle is essential in determining beauty, the growth pattern, treatment planning for skeletal class II and III patients, and age estimation in forensic medicine; it is averagely 128°±2.36° in males and 126°±2.41° in females [36]. Ohm and Silness found no significant difference in the size of the gonial angle between males and females [36]. Some studies reported an increase in gonial angle size with age, while others reported otherwise [37–40].

Studies on the esthetics of gonial angle are scarce, including nonstandardized ones on the male jaw angle [35]. Due to the importance of the jaw angle in beauty and the increasing demand for its surgical alteration, and in light of the lack of studies on female jaw angle as well as the mentioned shortcomings in the literature, this Perceptometric study is aimed at finding the range of pleasing frontal view and three-quarter oblique-view female intergonial width and gonial height comparatively from the perspectives of OMF surgeons, orthodontists, and laypeople of different ages and sexes. We also used for the first time multivariate as well as within-subject multivariable univariate statistical analyses to properly address the correlations among the variables. The goal was to test if different groups of judges would dissimilarly rate the set of Perceptometric images, and if so, which group would be more sensitive to anatomical changes. The study also examined simultaneously the potential role of the judges' sex and age on their esthetic preferences. The null hypotheses were a lack of any differences across the 3-rater groups, among different rater ages, between the sexes and across Perceptometric anatomical changes of the female photo model.

## 2. Materials and Methods

This was a prospective, psychometric, and diagnostic study on 4191 esthetic preferences of 127 individuals (judges) towards 33 Perceptometric facial images. All the raters and also the photo model agreed to participate after proper explanation. No personal identifiers were collected. The protocol and its ethics were approved by the Research Committee of the University (ethics code: IR.AJUMS.REC.1399.770).

### 2.1. Sample Size

The sample size of this study was calculated using G Power 3.1.9.2 software to be 32 in each of the 3 groups according to the parameters borrowed from a recent study on beauty factors [41], to obtain a 95% study power, assuming an alpha of 0.05, and using the following parameters: a standard deviation of 1.48 and precision of 0.303, maximum mean attractiveness score for 14° alteration of the gonial angle on the photograph of a female model to be 9.06 ± 1.19 by orthodontists, 7.33 ± 2.15 by OMF surgeons, and 7.53 ± 2.12 by laypeople, and the effect size of 0.57. The sample size was doubled-up in the case of laypeople who were much more available than specialists.

### 2.2. Original Photographs

After obtaining written informed consent from the photo model, frontal and three-quarter oblique view photographs were obtained from a young woman with a normal face, i.e., mesofacial, not brachyfacial, and not dolichofacial, and also without skeletal class II or III malocclusions. The female model had to be aged between 18 and 30 years, had to have class I skeletal relationship, had to have no asymmetry or craniofacial syndrome, and no history of facial cosmetic procedures.

### 2.3. Perceptometric Image Sets with Controlled Variable Morphologies

One examiner identified the zygion and gonion anthropometric landmarks and the mandibular plane inclination on both digital photographs, two experienced orthodontists then confirmed them. If there were any disagreement between specialists, it would be settled through discussion by the two orthodontists with a third one.

#### 2.3.1. Intergonial Width

**Figure 1:**
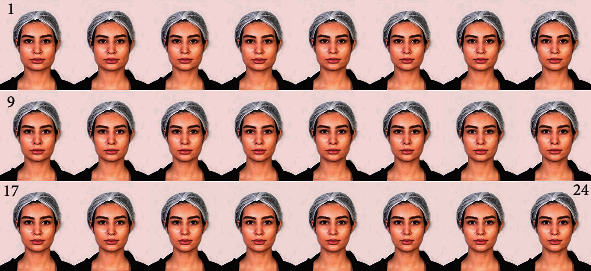
The frontal-view Perceptometric image set with serial decrease of the intergonial width. The intergonial width-to-interzygomatic width ratios are 116.6%, 114.7%, 112.8%, 110.9%, 108.99%, 107.07%, 105.15%, 103.2%, 101.31%, 99.39%, 96.35%, 95.5%, 92.96%, 91.2%, 89.57%, 87.8%, 86.03%, 84.04%, 82.12%, 80.21%, 78.29%, 76.37%, 74.45%, and 72.53% in images 1 to 24.

According to a renowned orthodontic textbook, the ideal gonion should be horizontally in line with the outer corner of the eye [42]. The original intergonial width of the photo model was 2 mm smaller than this ideal measurement. The original image was considered the image 16 in Figure 1. The intergonial width was altered using the Photoshop 2017 software (Adobe, San Jose, California, USA) on the frontal view photograph so that the ideal intergonial width [42] was reached. This was considered the ideal face and one of the middle images (image 15 in Figure 1).

Newer images were created by reducing or increasing the intergonial width, using Photoshop to obtain 24 images (Figure 1). Each increment was 2 mm, i.e., in each image, the intergonial width was 2 mm shorter than the previous image and 2 mm longer than the next image (Figure 1). The first and the last images had the widest and narrowest intergonial widths, respectively. The numbers of images before and after the ideal face were not similar, because after a limit of photograph manipulation, the images would look unrealistic and unusable.

The ratio of the intergonial width to the interzygomatic width (the IG : IZ ratio) was calculated for each image. In the Perceptometric frontal images 1 to 24, the IG : IZ ratios were, respectively, 116.6%, 114.7%, 112.8%, 110.9%, 108.99%, 107.07%, 105.15%, 103.2%, 101.31%, 99.39%, 96.35%, 95.5%, 92.96%, 91.2%, 89.57% (the ideal ratio suggested by a textbook), 87.8% (the original image), 86.03%, 84.04%, 82.12%, 80.21%, 78.29%, 76.37%, 74.45%, and 72.53% (Figure 1).

#### 2.3.2. Gonial Height

**Figure 2:**
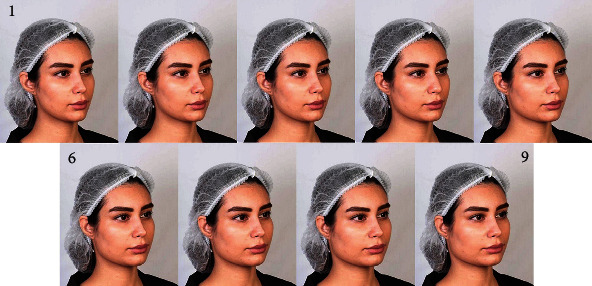
The three-quarter oblique-view Perceptometric image set with serial lowering of the gonion. In images 1 to 9, the gonion was, respectively, 4.5 mm above the mouth corner, at the level of mouth corner, 4.5 mm below the mouth corner, 9 mm, 13.5 mm, 18 mm, 22.5 mm, 27 mm, and 31.5 mm below the mouth corner.

The ideal gonion height was determined as being at the level of the mouth corner [35]. The original oblique three-quarter image taken from the photo model was the image 4 of Figure 2. On the three-quarter oblique view photograph, the location of gonion was gradually altered using Photoshop within the range of normal facial height to obtain 9 photographs (Figure 2). By increments of 4.5 mm, the height of gonion was both increased and reduced to develop 3 images before this image and 5 images after it. The probably ideal image [35], on which gonion was at the level of the mouth corner was the image 2 of Figure 2.

This way, the gonion in the Perceptometric three-quarter oblique images 1 to 9 would be positioned, respectively, 4.5 mm above the mouth corner, at the level of the mouth corner, 4.5 mm below it, 9 mm below it (the original image), 13.5 mm below it, 18 mm below it, 22.5 mm below it, 27 mm below it, and 31.5 mm below the mouth corner (Figure 2). Again, the numbers of images before and after the ideal face were not similar, as after a limit of photo manipulation, the images would look unrealistic.

### 2.4. Esthetic Assessments

#### 2.4.1. Raters

A total of 325 participants rated the images, but many of them did so only partially and left at least one question unanswered. All such participants were excluded; only those who had completely rated all the 33 images were kept as judges. Three groups of judges evaluated the photographs: 32 OMF surgeons, 33 orthodontists, and 62 laypeople.

#### 2.4.2. Questionnaire

Two orthodontists designed the questions of the electronic questionnaire, which included demographics as well as 33 esthetic questions corresponding to the 24 frontal images and 9 oblique images as explained. Using the online questionnaire, the 33 photographs were shown to the examiners. By showing each photograph to each examiner, he or she was requested to rate the attractiveness of each photograph using a 11-point Numeric Rating Scale (NRS, scores 0 to 10). They were instructed that lower scores meant less attractiveness and higher scores should be given to more pleasing faces. The participants were blinded to the original images of the photo model as well as the ideal images (suggested by the references [35, 42]).

#### 2.4.3. Questionnaire Reliability

The internal consistencies of the questionnaire items pertaining to the frontal and oblique view image sets were separately assessed using the Cronbach Alpha. It showed excellent internal consistencies for the intergonial width (Alpha = 0.987, *P* < 0.0005) and gonial height questions (Alpha = 0.972, *P* < 0.0005).

### 2.5. Statistical Analysis

The software in use was SPSS 25 (IBM, Armonk, NY, USA). Descriptive statistics were calculated for each of the 33 variables (24 intergonial questions and 9 gonial height questions) in each of the 3 different groups. The ages and sexes of examiners in different groups were compared using an independent-sample *t*-test and a chi-square test, respectively. A two-way multivariate analysis of covariance (MANCOVA) and follow-up univariate analyses of covariance (ANCOVA) were performed to assess the effects of the variables' expertise, age, and sex of the judges on their esthetic preferences. The significant MANCOVA/ANCOVA results were followed by a Bonferroni post hoc test. A Pearson correlation coefficient was used to examine the correlations between the overall scores of the 127 judges given to the frontal-image questionnaires (the average of all 24 images) with the overall scores of the same persons given to the three-quarter-view questionnaires (the average of all 9 images). In other words, this coefficient was used to test if for example, a particular judge had given higher scores to the frontal image set, whether or not the same judge would give also higher scores to the 3/4 image set.

The sensitivity of the judges to the Perceptometric serial anatomic alterations (i.e., the extent of changes in esthetic preferences as a function of photogrammetric stimuli) was assessed using a 2-way repeated-measures analysis of covariance (RM-ANCOVA) followed by a Bonferroni post hoc test. This analysis was used to assess the differences in scores of different images within each Perceptometric set; at the same time, it was used to examine the effects of the expertise of the examiners (OMFS, orthodontist, and laypeople), their sex, and their age on the changes happened to their esthetic scores given to the images as a function of the serial anatomic (Perceptometric) changes.

The level of significance was set at 0.05 for all statistical tests, except for those between subject ANCOVAs, which followed the two-way MANCOVAs (and not for the 2-way RM-ANCOVAs). For such follow-up, ANCOVAs performed on the 24-intergonial width comparisons, the level of significance was adjusted to 0.0021, using the Bonferroni method. For those follow-up, ANCOVAs used for the analysis of the 9 gonial height questions, the level of significance was adjusted to 0.0056, using the Bonferroni method.

## 3. Results

The judges included 63 men and 64 women. The numbers of males and females in different groups were, respectively, 21 male and 11 female OMF surgeons, 14 male and 19 female orthodontists, and 28 male and 34 female laypeople. The sex distribution of different groups did not differ significantly with each other according to the chi-square test (*P* = 0.108). The mean (SD) age of the sample was 35.72 ± 9.453 years. The mean ages of the examiners were 37.41 ± 7.97 years (min: 24, max: 61) in OMF surgeons, 35.73 ± 5.77 years (min: 29, max: 54) in orthodontists, and 34.85 ± 11.52 years (min: 14, max: 57) in laypeople. According to the independent-samples *t*-test, there was no significant difference in ages of different groups (*P* = 0.467). The mean age of females and males were, respectively, 33.22 ± 8.65 years (min: 14, max: 55) and 38.27 ± 9.62 years (min: 22, max: 61). The difference between ages of males and females was significant (*P* = 0.002).

### 3.1. Intergonial Width

**Figure 3:**
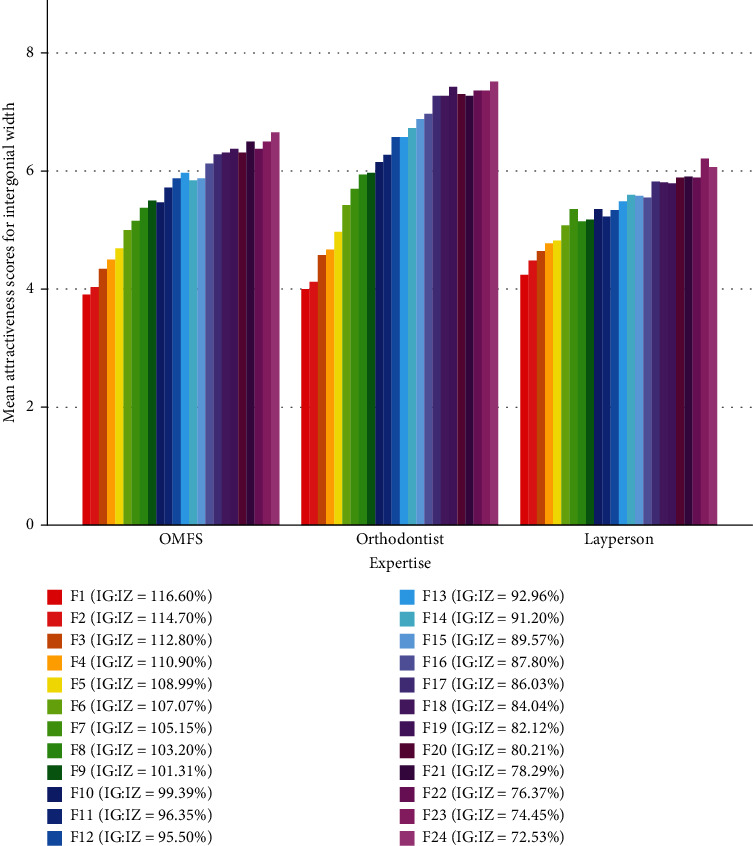
Mean attractiveness scores given to different intergonial widths depicted on frontal-view Perceptometric photos.

All groups favored smaller intergonial widths (Table 1, Figure 3), with orthodontists showing a steeper preference slope (Figure 3).

The overall means (SD) of the esthetic scores given by all the 127 raters to the images 1 to 24 were, respectively, 4.09 ± 1.83, 4.28 ± 1.89, 4.55 ± 1.93, 4.68 ± 1.89, 4.83 ± 1.96, 5.15 ± 1.87, 5.39 ± 1.96, 5.41 ± 1.87, 5.46 ± 2.02, 5.59 ± 1.95, 5.62 ± 1.96, 5.80 ± 1.90, 5.89 ± 1.91, 5.95 ± 1.97, 5.99 ± 1.93, 6.06 ± 1.99, 6.31 ± 1.87, 6.31 ± 1.99, 6.36 ± 1.99, 6.36 ± 1.99, 6.41 ± 1.91, 6.39 ± 1.97, 6.58 ± 1.86, and 6.59 ± 1.98, marking the last image as the most beautiful one.

Separately assessing, OMF surgeons and orthodontists gave the maximum attractiveness scores regarding intergonial width to photograph 24 (Figure 3) with an IG : IZ ratio of 72.53%. In comparison, the laypeople gave the maximum score to photograph 23 (Figure 1) with an IG : IZ ratio of 74.45%. OMF surgeons and orthodontists gave the minimum score of attractiveness regarding intergonial width to photograph 1 (Figure 1) with an IG : IZ ratio of 116.6%; whereas, the laypeople gave the minimum score to photograph 3 (Figure 1) with an IG : IZ ratio of 112.8%.

#### 3.1.1. Factors Affecting the Overall Preferences of Surgeons, Orthodontists, and Laypeople

The 2-way MANCOVA's results indicated that the judges' age (*P* = 0.322) and sex (*P* = 0.163) had no significant effect on their esthetic preferences regarding the intergonial width. However, the effect of expertise was significant (*P* = 0.038). The interaction of expertise and sex was nonsignificant (*P* = 0.644).

The follow-up ANCOVAs' *P* values (*α* = 0.0021) pertaining to the comparisons of the esthetic preferences of 3 “expertise” groups towards the intergonial width questions 1 to 24 across the 3 groups were, respectively, 0.8357, 0.6959, 0.9418, 0.9787, 0.8839, 0.6030, 0.6066, 0.1207, 0.1151, 0.1239, 0.0252, 0.0070, 0.0119, 0.0197, 0.0046, 0.0014, 0.0005, 0.0014, 0.0003, 0.0025, 0.0015, 0.0013, 0.0114, and 0.0014. In other words, there were significant differences across the 3 “expertise” groups in terms of each of the images 16 (IG : IZ = 87.80%), 17 (IG : IZ = 86.03%), 18 (IG : IZ = 84.04%), 19 (IG : IZ = 82.12%), 20 (IG : IZ = 80.21%), 21 (IG : IZ = 78.29%), 22 (IG : IZ = 76.37%), and 24 (IG : IZ = 72.53%).

The Bonferroni post hoc test showed that in any of the mentioned images (i.e., 16, 17, 18, 19, 20, 21, 22, and 24), the only significant pairwise comparison existed between the scores of orthodontists and laypeople (Table 2). There was no significant difference between OMF surgeons and orthodontists or between surgeons and laypeople (Table 2).

#### 3.1.2. Preference-Change Slopes and the Esthetically Pleasant Range

**Figure 4:**
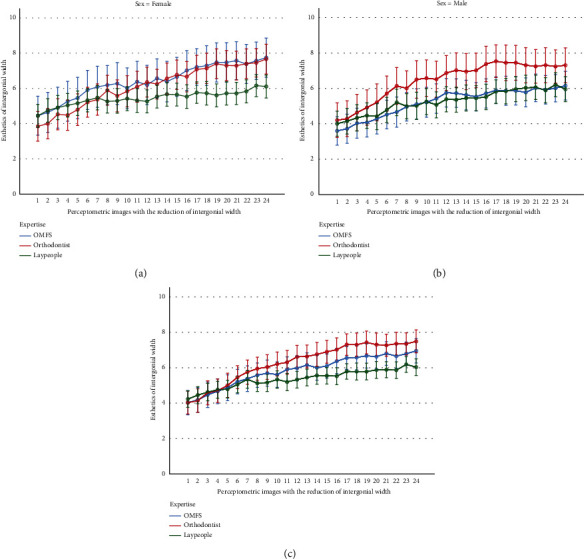
Mean attractiveness scores (and 95% confidence intervals) for the 24 frontal-view Perceptometric images related to the intergonial width changes from the perspective of females (a), males (b), and both sexes combined (c). The intergonial width-to-interzygomatic width ratios in images 1 to 24 are, respectively, 116.6%, 114.7%, 112.8%, 110.9%, 108.99%, 107.07%, 105.15%, 103.2%, 101.31%, 99.39%, 96.35%, 95.5%, 92.96%, 91.2%, 89.57%, 87.8%, 86.03%, 84.04%, 82.12%, 80.21%, 78.29%, 76.37%, 74.45%, and 72.53%.

The 2-way RM-ANCOVA showed that there was a significant difference across the preference scores given to the 24 images (*P* < 0.0000005, Figure 4). The Bonferroni post hoc test showed that most pairwise comparisons between the esthetic scores of distant images were significant (Table 3). In this regard, there was a significant difference in the esthetic scores given to the image that would be considered ideal according to the textbook reference [42] (image 15 of Figure 1) with the image that was actually considered the best according to our data (image 24 of Figure 1, Table 3). According to the Bonferroni test, there was no significant difference between the esthetic score of image 24 (the one with the best results) versus the esthetic scores of images 17 to 23 (Table 3), meaning that images 17 to 23 would be similarly pleasant to many observers. Image 16 had a marginally significant difference with image 24 (range of esthetically pleasing images: 17 to 24, Table 3).

The slopes of changes in esthetic preferences across the 24 images differed by age (*P* = 0.020 for the interaction of age and the serialized imaging). The interaction of serialized preferences with sex was nonsignificant (*P* = 0.435). However, the interaction of serialized preferences with the factor “expertise” was significant (*P* < 0.0000005), meaning that different experts (orthodontist, surgeons, and laypeople) had different slopes of “esthetic preference changes” across the 24 images (Figure 4). The interaction between the serialized imaging by expertise by sex was insignificant (*P* = 0.203).

The effects of age (*P* = 0.314) and sex (*P* = 0.291) were insignificant. However, the effect of expertise was significant (*P* = 0.037). The Bonferroni post hoc test showed that the only significant pairwise comparison existed between orthodontists and laypeople (*P* = 0.034) but not between orthodontists and surgeons (*P* = 0.838) or between surgeons and laypeople (*P* = 0.654). The interaction of sex by expertise was insignificant (*P* = 0.192).

### 3.2. Gonial Height

**Figure 5:**
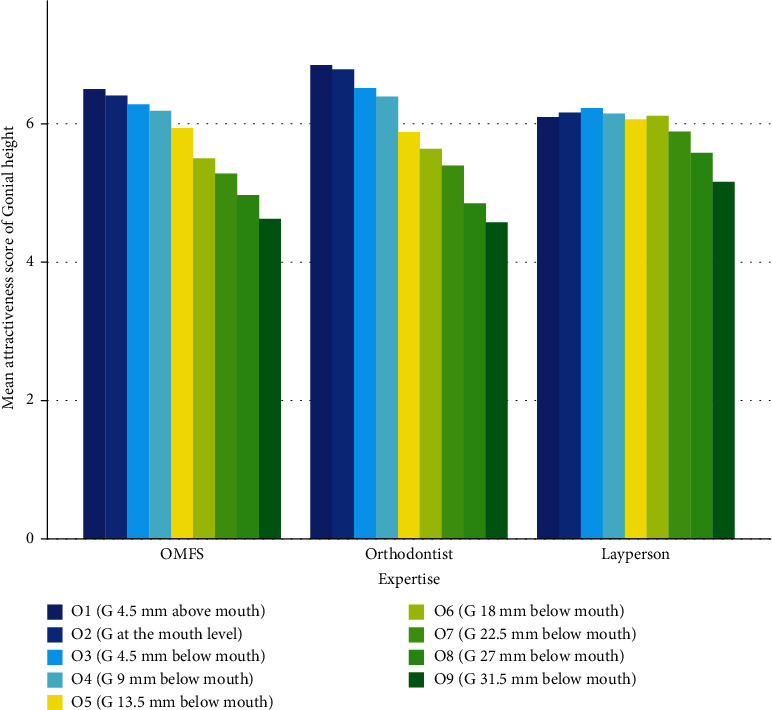
Mean attractiveness scores given to different gonial heights visible on Perceptometric three-quarter oblique-view images.

All groups favored mostly gonions almost at the mouth corner, with laypeople showing an apparent less steep slope of change in the scores given to vertical gonial anatomic modifications (Table 4, Figure 5).

The overall mean (SD) esthetic values given by all the 127 raters to images 1 to 9 were, respectively, 6.39 ± 1.89, 6.39 ± 1.72, 6.31 ± 1.75, 6.22 ± 1.93, 5.98 ± 2.06, 5.83 ± 2.12, 5.61 ± 2.17, 5.24 ± 2.32, and 4.87 ± 2.38, marking images 1 and 2 as the most beautiful ones.

Evaluating separately, it was seen that OMF surgeons and orthodontists gave the maximum score to photograph 1 (Figure 5) with a gonial height 4.5 mm above the mouth corner, while the laypeople gave the maximum score to photograph 3 (Figure 5) with a gonial height 4.5 mm below the mouth corner. All three groups gave the minimum attractiveness score regarding the gonial height to photograph 9 (Figure 5) with a gonial height 31.5 mm below the mouth corner.

#### 3.2.1. Factors Affecting the Overall Preferences of Orthodontists, Surgeons, and Laypeople

The 2-way MANCOVA's results showed that age (*P* = 0.325) and sex (*P* = 0.114) had no significant influence on the esthetic preferences of the raters regarding the gonial height. However, the effect of expertise was significant (*P* = 0.017). The interaction of expertise and sex was nonsignificant (*P* = 0.621).

The follow-up ANCOVAs' *P* values (*α* = 0.0056) regarding the comparisons of the esthetic preferences of 3 “expertise” groups towards the gonial height questions 1 to 9 became all nonsignificant (*P* ≥ 0.111). The Bonferroni post hoc test was not performed.

#### 3.2.2. Preference-Change Slopes and the Esthetic Zone

**Figure 6:**
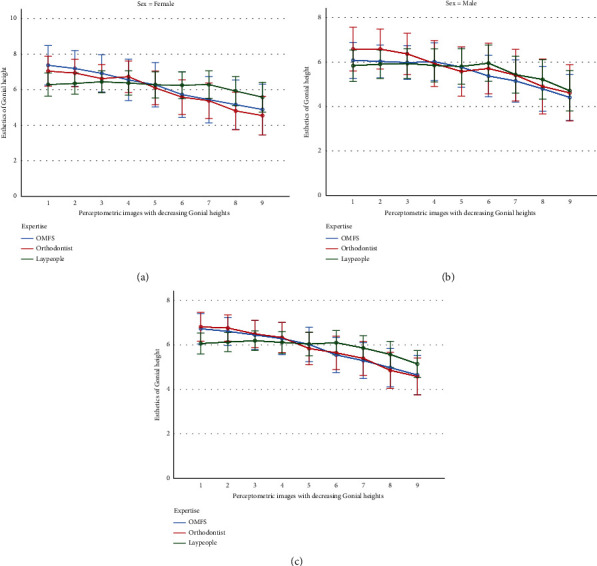
Mean attractiveness scores (and 95% confidence intervals) for the 9 three-quarter-view Perceptometric images related to the gonial height changes from the perspective of females (a), males (b), and both sexes combined (c). In images 1 to 9, the gonion was, respectively, 4.5 mm above the mouth corner, at the level of the mouth corner, 4.5 mm below the mouth corner, 9 mm, 13.5 mm, 18 mm, 22.5 mm, 27 mm, and 31.5 mm below the mouth corner.

According to the 2-way RM-ANCOVA, there was a significant difference across the preference scores given to the 9 images (*P* < 0.0000005, Figure 6). The Bonferroni post hoc test showed significant differences between the far images (Table 5). There was no significant difference between the image with the ideal standards of gonial height according to a previous study [35] (image 2 of Figure 2) with the image with the highest esthetic scores in this study (image 1 of Figure 2, Table 5). The esthetic scores of images 1 and 2 (as the ones with the greatest overall esthetic score) were not significantly different from the esthetic scores of images 3 and 4 (Table 5), meaning that the range of esthetically pleasing zone were images 1 to 4.

The slopes of changes in esthetic preferences across the 9 images differed by age (*P* = 0.009 for the interaction of age and the serialized imaging). The interaction of serialized preferences with sex was nonsignificant (*P* = 0.371). However, the interaction of serialized preferences with the factor “expertise” was significant (*P* = 0.00004), meaning that different experts (orthodontist, surgeons, and laypeople) had different slopes of “esthetic preference changes” across the 9 images (Figure 6). The interaction between the serialized imaging by expertise by sex was insignificant (*P* = 0.322).

The role of age (*P* = 0.862), sex (*P* = 0.185), and expertise (*P* = 0.982) was insignificant. The interaction of sex by expertise was insignificant as well (*P* = 0.887).

### 3.3. Correlations between Both Perceptometric Image Sets

The Pearson correlation coefficient showed that there was a strong positive correlation between the average scores given to the frontal images and the average scores given to the 3/4 oblique images (*R* = 0.719, *P* < 0.00000005), meaning that if a judge had given a high score to the one of the two image sets, there would be a strong tendency for the same judge to give higher scores also to the other of the set. In other words, many judges tended to give higher esthetic scores regardless of the set of photographs in question, while many others tended to give lower esthetic scores to both sets, again regardless of the photographic set.

## 4. Discussion

The first step in the assessment of facial ratios is to examine the frontal view. In the frontal view, the gonial angle is influenced by the skeletal facial pattern, volume of the masseteric muscle, and skin coverage. The facial index (facial height/width ratio) determines the face type, and assessment of facial height should include the assessment of facial width [42, 43]. According to the textbooks [42, 43], an ideally proportional face is made of the equal fifths of central, medial, and lateral, based on the inner and outer canthi of the eyes. The ideal facial form might be when a vertical line from the inner canthus coincides with the ala of the nose base, and when a vertical line from the outer eye canthi touches the gonial angles [42]. However, our results in terms of attractive female gonial angles was not in line with the textbook [42]. Various reasons can be cited for this difference. Firstly, the textbook suggestion was not based on evidence-based research, but merely based on personal and subjective assumptions and opinions. Therefore, that particular part of the textbook should not be trusted as some hardcore scientific fact, but merely regarded as some suggestion. Moreover, facial beauty might be affected by the ethnicity of the person and the culture or ethnicity of the judges, as well as numerous other socioeconomic, demographic, and educational determinants [7–9, 17, 21, 28, 29, 42]. Not to mention that other factors as well can affect the judgement of esthetics, even technical aspects such as the camera lenses or sensors in use [44, 45]. No similar study existed in this regard for us to compare our results, and future research should verify and test our findings in other populations. According to the personal opinion of Aiache [43], the ideal intergonial width should be equal to or 10% smaller than the interzygomatic width in the frontal view. The bitemporal width is equal to the intergonial width [43]. In a study by Mommaerts [35], esthetics of nonstandardized photographs of models or celebrities were surveyed; the author found that intergonial width equal to facial width was considered ideal for men. In the present study, OMF surgeons and orthodontists gave the maximum score to the IG : IZ ratio = 72.53%, while this ratio was 74.45% for the laypeople.

In contrast to the classic definition of a flawless face with equal vertical thirds, the contemporary definition of facial esthetics emphasizes a more significant inferior facial third and a square jaw, particularly in males [42, 43]. Strong jaw angles and chins are perceived as masculine features; hence, surgical feminization of female or transgender faces might need reduction of borders and angles of the mandibular base and decreasing chin dimensions [46]. On the other hand, deficient mandibular angles may be augmented with polyethylene implants suitably modeled and attached to the mandible using extraoral incisions [47]. Standard and custom jaw angle implants seem promising solutions to many jawline corrections [48]. These comprise lateral widening and vertical lengthening, which are not pure and isolated categories; in other words, widening implants may also subtly lengthen the vertical dimension, and lengthening ones may also widen the jaw angle to some degree [48]. Another way of defining the jaw angle (and also straightening the jaw contour) is using dermal fillers [49]. These are consisted of various materials such as calcium hydroxyapatite with integral lidocaine or without it, a hybrid mixture of calcium hydroxyapatite and hydroxyapatite, or a prime hydroxyapatite filler, contingent on the preferred cosmetic result and the patient's request [49]. Furthermore, jawline contouring is possible through the use of custom-built 3D-constructed titanium implants [50]. Our findings were in line with those of Aiache [43]; this indicates higher attractiveness of smaller lower facial width in women. These findings also agree with Mommaerts [35] who showed tapered or trapezoidal are more desirable for female forms [35]. At present, there is a growing demand among patients to increase their intergonial width. Several techniques are used to achieve this goal, such as the clockwise rotation of the condyles in a bilateral sagittal split osteotomy and the placement of titanium implants [51–53]. It should be noted, however, that in females, such augmenting surgical approaches may actually make the patient less esthetically appealing. This is because the ideal intergonial widths chosen by the surgeons, orthodontists, and laypeople were almost the same, all favoring narrower intergonial widths. This finding indicates that wider faces are less attractive to all three groups of raters. According to the concept of lower third analysis, it is suggested (without any scientific study) that an appropriate mandibular width for a female is narrower than zygomatic width [54].

According to the present results, gonial height at the level of the mouth corner or a close distance is probably attractive to the observers. This finding can help the clinicians performing cosmetic procedures. In general, the scoring by OMF surgeons and orthodontists was similar but, overall, different from the scoring by the laypeople. To assess this more on the level of each of the 9 images alone, more data were needed. Moreover, we observed that the judges' profession could affect their sensitivity to Perceptometric changes. According to some authors, laypeople may judge facial esthetics like dental professionals [35, 55–57]; moreover, in the case of generally beautiful faces, the greater education and experience of specialists and orthodontists in comparison to laypeople might not necessarily count [58]. Some other studies point to similarities between experts and laypeople in the case of some variables but differences between these groups in terms of some other parameters [6]. In the present study, it was shown that the expertise of dental professionals may matter in terms of the overall scores and also in terms of their tolerance or sensitivity to the Perceptometric changes. Especially, the overall differences became more vivid for orthodontists versus laypeople when evaluating the frontal images with attractive intergonial widths. The differences across the esthetic preferences of different professions were not significant between the laypeople and OMF surgeons, and between OMF surgeons and orthodontists; however, in the case of the intergonial width, the difference was significant between orthodontists and laypeople to highlight the role of knowledge, experience, and training in this respect. Perhaps, such results can be explained in terms of the stronger focus of orthodontists to the facial beauty and the lower esthetic education of laypeople, making them rather indifferent to the anatomic changes. Moreover, such results can be explained by a longer duration of time studying facial beauty; this latter explanation also may hold for the effect of age observed in this study on the sensitivity of the judges to Perceptometric changes. Our findings indicated that males and females might perceive the jaw angle beauty similarly. This finding was similar to studies on other esthetic factors [6]. However, we could not find any studies directly similar to ours, in order to compare our results with. The observed differences in the professionals' and laypersons' perspectives of the jaw angle beauty raise questions regarding the clinical implications of this study. Which one of the esthetic preferences found in our study should clinicians refer to? Of course, each group of experts should prioritize the preferences of their own specialty. But above them all, the preferences of laypeople seem to matter the most, because most of the time, patients are themselves laypersons and judged in the society by laypeople. Therefore, it seems that the preferences of laypeople should have the highest priority for treatment.

We observed that there is a tendency to give higher or lower scores to the Perceptometric sets, meaning that someone who had given a higher average score to the frontal image set would also give higher marks to the 3/4 image set. This may have at least two reasons: the first one is that the images were not limited to the gonial angle area; they had the whole face of the model, in which all facial parts were visible and thus affecting the judgement of beauty. In other words, for example, a given judge may perceive the female model herself (as a whole) as a very attractive person; this way, the average esthetic scores given by that judge would elevate, regardless of the image set in question. While another judge may perceive the model as mediocre, causing the average scores to drop, again regardless of the gonial angle or the image set. The second reason may be that it is possible that some judges may have more stringent criteria for the perception of facial beauty, causing them to give lower scores, again regardless of the set of images or even the gonial angle itself.

This study had some limitations. A question is that now that the most attractive images were at one of the two ends of the image sets (and not somewhere in between), was it possible that by some more alterations (e.g., narrowing the jaw angle width more than image #24), we obtain even higher esthetic results? For addressing such a question, a wider range of anatomical changes were necessary, which was not provided in this research due to serious limitations a long questionnaire would impose. Therefore, at the beginning of the study, the researchers who chose the photos came to the conclusion that the images with a width less than that on image 24 would create unpleasant, inharmonious, and unnatural faces. Therefore, to avoid extending the questionnaire, such excessively narrow images were removed. But, it is possible to examine even narrower gonial widths in future studies to see at which point, narrowing the gonial angle width would stop to result in higher esthetic scores. The same can be said for the gonial angle height. Another limitation is the lack of evaluating both vertical and horizontal dimensions together. We can see visually that each set of images has a slope of attractiveness. It can be inferred visually that there may be some point at which the intergonial width reaches its most beautiful state, and at the same time, the gonial height reaches its most beautiful state. A question is that, if there is a point where both of the vertical and horizontal positions of the gonial angle will become ideal simultaneously, what would be that particular 2D position (on both the vertical and horizontal axes)? The other question is that, if we can be certain that such a point where both the variables reach their maximum beauty can be considered as the most attractive one. The answer is, we cannot conclude that for sure. For knowing the answer to the second question, we need a third set of Perceptometric images, on which both the intergonial width and gonial height are manipulated simultaneously (on both vertical and horizontal axes), in order to see how their combination looks attractive. Therefore, this study was unable to answer the latter question; future studies with images changing both variables simultaneously are needed to assess this. Nevertheless, such studies will have a very large number of images (such as 9 vertical changes × 24 horizontal changes = 216 photographs) making them very difficult to conduct. Of course, the number of images within the set can be reduced by increasing the jumps in the gonial angle positions, but even with two sets of 9 anatomical changes each, a total of 81 images would be needed, which will make the questionnaire very long and tiresome. The same limitation disallowed us to include also a male model, or models from various races. This is because adding each model would actually increase the number of photographs by 31, severely discouraging potential judges from participating. Moreover, by increasing the number of images, the accuracy of judges' esthetic scores would drop. Therefore, we were limited to study only females of one race. Future studies are warranted to assess also males and other ethnic backgrounds.

## 5. Conclusion

It could be concluded that narrower female jaw angles may be unanimously more desirable by orthodontists, OMF surgeons, and laypeople, regardless of the judges' sex or age. The range of esthetically acceptable IG : IZ ratio was 72.53% to 86.03%. The education of observers may affect their perception of beauty. Female jaw angles positioned almost at the level of the mouth corner would be more beautiful according to judges of any sex or age. The range of esthetically acceptable gonial height was from 4.5 mm above the mouth corner to 9 mm below the mouth corner. The judges' expertise can influence their perception.

## Figures and Tables

**Table 1:** Descriptive statistics for the scores given by the three groups of raters to photographs with altered intergonial widths.

Image	Sex	OMFS					Orthodontists					Laypersons				
		*N*	Mean	SD	Min	Max	*N*	Mean	SD	Min	Max	*N*	Mean	SD	Min	Max
F1	Female	11	4.45	1.21	3	7	19	3.84	2.69	1	10	34	4.41	1.79	0	10
	Male	21	3.62	0.92	2	5	14	4.21	1.72	2	7	28	4.04	1.97	1	9
	Both	32	3.91	1.09	2	7	33	4.00	2.30	1	10	62	4.24	1.87	0	10

F2	Female	11	4.64	1.36	3	7	19	4.00	2.75	0	10	34	4.76	1.67	2	10
	Male	21	3.71	1.49	0	6	14	4.29	1.59	2	7	28	4.14	2.01	1	9
	Both	32	4.03	1.49	0	7	33	4.12	2.30	0	10	62	4.48	1.84	1	10

F3	Female	11	4.91	1.30	4	7	19	4.53	2.52	1	9	34	4.88	1.75	0	10
	Male	21	4.05	1.56	1	7	14	4.64	1.55	2	7	28	4.36	2.30	1	9
	Both	32	4.34	1.52	1	7	33	4.58	2.14	1	9	62	4.65	2.02	0	10

F4	Female	11	5.27	1.10	4	7	19	4.47	2.39	0	10	34	5.00	1.86	0	10
	Male	21	4.10	1.70	0	7	14	4.93	1.69	2	7	28	4.50	1.99	1	9
	Both	32	4.50	1.61	0	7	33	4.67	2.10	0	10	62	4.77	1.92	0	10

F5	Female	11	5.45	1.13	4	7	19	4.79	2.49	1	9	34	5.15	2.02	0	10
	Male	21	4.29	1.76	0	7	14	5.21	1.76	2	7	28	4.43	1.97	1	9
	Both	32	4.69	1.65	0	7	33	4.97	2.19	1	9	62	4.82	2.01	0	10

F6	Female	11	5.91	1.22	4	7	19	5.21	2.35	2	10	34	5.32	1.89	0	10
	Male	21	4.52	1.66	1	7	14	5.71	1.38	2	7	28	4.79	1.97	1	9
	Both	32	5.00	1.65	1	7	33	5.42	1.98	2	10	62	5.08	1.93	0	10

F7	Female	11	6.09	1.22	4	7	19	5.37	2.41	1	10	34	5.47	2.03	0	10
	Male	21	4.67	1.77	1	8	14	6.14	1.79	2	8	28	5.21	1.93	1	9
	Both	32	5.16	1.72	1	8	33	5.70	2.17	1	10	62	5.35	1.98	0	10

F8	Female	11	6.18	1.08	4	7	19	5.89	2.26	3	10	34	5.29	1.88	0	10
	Male	21	4.95	1.83	1	8	14	6.00	1.52	4	8	28	4.96	1.91	1	9
	Both	32	5.38	1.70	1	8	33	5.94	1.95	3	10	62	5.15	1.89	0	10

F9	Female	11	6.27	1.10	4	7	19	5.58	2.59	1	10	34	5.32	2.01	0	10
	Male	21	5.10	1.87	1	8	14	6.50	1.56	4	9	28	5.00	2.04	1	9
	Both	32	5.50	1.72	1	8	33	5.97	2.23	1	10	62	5.18	2.01	0	10

F10	Female	11	6.00	1.10	4	7	19	5.84	2.36	1	10	34	5.47	2.02	0	10
	Male	21	5.19	1.97	1	8	14	6.57	1.65	4	9	28	5.21	1.87	1	9
	Both	32	5.47	1.74	1	8	33	6.15	2.09	1	10	62	5.35	1.94	0	10

F11	Female	11	6.36	1.21	4	8	19	6.11	2.33	1	10	34	5.41	1.91	0	10
	Male	21	5.38	1.72	2	8	14	6.50	2.07	2	10	28	5.00	1.96	1	9
	Both	32	5.72	1.61	2	8	33	6.27	2.20	1	10	62	5.23	1.93	0	10

F12	Female	11	6.18	1.25	4	8	19	6.37	2.24	2	10	34	5.35	1.92	0	10
	Male	21	5.71	1.49	2	8	14	6.86	1.79	3	10	28	5.32	1.98	1	9
	Both	32	5.88	1.41	2	8	33	6.58	2.05	2	10	62	5.34	1.93	0	10

F13	Female	11	6.55	1.21	4	8	19	6.26	2.33	1	10	34	5.68	1.95	1	10
	Male	21	5.67	1.43	2	9	14	7.00	1.80	3	9	28	5.25	1.94	1	9
	Both	32	5.97	1.40	2	9	33	6.58	2.12	1	10	62	5.48	1.94	1	10

F14	Female	11	6.36	1.12	4	8	19	6.58	2.12	3	10	34	5.79	2.14	0	10
	Male	21	5.57	1.57	1	9	14	6.93	1.73	3	9	28	5.36	2.11	1	9
	Both	32	5.84	1.46	1	9	33	6.73	1.94	3	10	62	5.60	2.12	0	10

F15	Female	11	6.64	0.81	5	8	19	6.79	2.04	3	10	34	5.76	2.03	0	10
	Male	21	5.48	1.60	2	9	14	7.00	1.80	3	9	28	5.36	1.99	1	9
	Both	32	5.88	1.48	2	9	33	6.88	1.92	3	10	62	5.58	2.00	0	10

F16	Female	11	7.00	1.10	5	9	19	6.68	2.06	1	10	34	5.65	2.03	0	10
	Male	21	5.67	1.71	2	9	14	7.36	1.78	3	10	28	5.43	2.04	1	9
	Both	32	6.13	1.64	2	9	33	6.97	1.94	1	10	62	5.55	2.02	0	10

F17	Female	11	7.18	1.08	5	9	19	7.11	2.00	3	10	34	5.94	1.86	3	10
	Male	21	5.81	1.50	2	9	14	7.50	1.45	5	10	28	5.68	2.02	1	9
	Both	32	6.28	1.51	2	9	33	7.27	1.77	3	10	62	5.82	1.92	1	10

F18	Female	11	7.27	1.10	5	9	19	7.16	2.06	2	10	34	5.85	2.00	0	10
	Male	21	5.81	1.86	2	9	14	7.43	1.55	5	10	28	5.75	2.08	1	9
	Both	32	6.31	1.77	2	9	33	7.27	1.84	2	10	62	5.81	2.02	0	10

F19	Female	11	7.45	1.04	5	9	19	7.42	1.74	3	10	34	5.74	2.03	0	10
	Male	21	5.81	1.66	2	9	14	7.43	1.83	4	10	28	5.86	2.12	1	9
	Both	32	6.38	1.66	2	9	33	7.42	1.75	3	10	62	5.79	2.06	0	10

F20	Female	11	7.45	1.04	5	9	19	7.32	1.89	2	10	34	5.85	1.99	0	10
	Male	21	5.71	1.71	2	9	14	7.29	1.86	4	10	28	5.93	2.14	1	10
	Both	32	6.31	1.71	2	9	33	7.30	1.85	2	10	62	5.89	2.04	0	10

F21	Female	11	7.55	1.13	5	9	19	7.32	1.45	4	10	34	5.88	2.13	0	10
	Male	21	5.95	1.80	2	10	14	7.21	1.58	4	9	28	5.93	1.96	1	9
	Both	32	6.50	1.76	2	10	33	7.27	1.48	4	10	62	5.90	2.04	0	10

F22	Female	11	7.36	1.21	5	9	19	7.42	1.74	2	10	34	5.97	2.10	0	10
	Male	21	5.86	1.65	2	9	14	7.29	1.77	3	9	28	5.79	2.06	1	10
	Both	32	6.38	1.66	2	9	33	7.36	1.73	2	10	62	5.89	2.07	0	10

F23	Female	11	7.55	1.13	6	9	19	7.47	1.50	4	10	34	6.29	1.77	3	10
	Male	21	5.95	1.53	2	9	14	7.21	2.42	2	10	28	6.11	2.01	1	10
	Both	32	6.50	1.59	2	9	33	7.36	1.92	2	10	62	6.21	1.87	1	10

F24	Female	11	7.73	1.10	6	9	19	7.68	1.63	3	10	34	6.24	1.81	3	10
	Male	21	6.10	1.61	2	9	14	7.29	2.43	2	10	28	5.86	2.17	1	10
	Both	32	6.66	1.64	2	9	33	7.52	1.99	2	10	62	6.06	1.97	1	10

F: frontal-view image; SD: standard deviation; Min: minimum; Max: maximum.

**Table 2:** The results of the Bonferroni post hoc test, comparing the esthetic scores given by the 3 rater groups to each of the questions with a significant ANCOVA.

Image	I	J	Difference (I-J)	SE	*P*	95% CI	
F16 (IG : IZ = 87.80%)	OMFS	Orthodontist	-0.65	0.49	0.5421	-1.83	0.53
	OMFS	Laypeople	0.84	0.43	0.1546	-0.20	1.88
	Orthodontist	Laypeople	1.50	0.41	**0.0012**	0.50	2.50

F17 (IG : IZ = 86.03%)	OMFS	Orthodontist	-0.75	0.45	0.2826	-1.84	0.33
	OMFS	Laypeople	0.76	0.39	0.1673	-0.20	1.72
	Orthodontist	Laypeople	1.52	0.38	**0.0003**	0.60	2.44

F18 (IG : IZ = 84.04%)	OMFS	Orthodontist	-0.72	0.49	0.4320	-1.90	0.47
	OMFS	Laypeople	0.79	0.43	0.2014	-0.25	1.84
	Orthodontist	Laypeople	1.51	0.41	**0.0011**	0.51	2.51

F19 (IG : IZ = 82.12%)	OMFS	Orthodontist	-0.76	0.48	0.3467	-1.91	0.40
	OMFS	Laypeople	0.89	0.42	0.1097	-0.13	1.91
	Orthodontist	Laypeople	1.65	0.40	**0.0003**	0.66	2.63

F20 (IG : IZ = 80.21%)	OMFS	Orthodontist	-0.68	0.48	0.4880	-1.85	0.49
	OMFS	Laypeople	0.75	0.43	0.2404	-0.28	1.78
	Orthodontist	Laypeople	1.43	0.41	**0.0020**	0.44	2.42

F21 (IG : IZ = 78.29%)	OMFS	Orthodontist	-0.47	0.46	0.9393	-1.59	0.65
	OMFS	Laypeople	0.91	0.41	0.0799	-0.07	1.90
	Orthodontist	Laypeople	1.38	0.39	**0.0018**	0.43	2.33

F22 (IG : IZ = 76.37%)	OMFS	Orthodontist	-0.70	0.48	0.4304	-1.86	0.46
	OMFS	Laypeople	0.79	0.42	0.1889	-0.23	1.81
	Orthodontist	Laypeople	1.49	0.40	**0.0010**	0.51	2.48

F24 (IG : IZ = 72.53%)	OMFS	Orthodontist	-0.54	0.48	0.7917	-1.70	0.62
	OMFS	Laypeople	0.92	0.42	0.0944	-0.11	1.94
	Orthodontist	Laypeople	1.46	0.41	**0.0014**	0.47	2.44

F: frontal view; SE: standard error; CI: confidence interval. Significant *P* values in bold.

**Table 3:** The Bonferroni test results comparing the esthetic scores of the 24 images related to intergonial width changes.

Image I	Image J	Difference (I-J)	SE	*P*	95% CI	
2	1	0.16	0.07	1.0	-0.09	0.42

3	1	0.47	0.09	**0.0007**	0.10	0.83
	2	0.30	0.09	0.1731	-0.03	0.63

4	1	0.62	0.11	**< 0.00005**	0.19	1.04
	2	0.45	0.10	**0.0019**	0.08	0.82
	3	0.15	0.09	1.0	-0.19	0.49

5	1	0.79	0.12	**< 0.00005**	0.32	1.27
	2	0.63	0.11	**< 0.00005**	0.21	1.05
	3	0.33	0.09	0.0650	-0.01	0.66
	4	0.18	0.09	1.0	-0.17	0.52

6	1	1.15	0.13	**< 0.00005**	0.66	1.65
	2	0.99	0.11	**< 0.00005**	0.55	1.42
	3	0.69	0.09	**< 0.00005**	0.32	1.05
	4	0.54	0.09	**< 0.00005**	0.17	0.90
	5	0.36	0.08	**0.0018**	0.06	0.65

7	1	1.40	0.14	**< 0.00005**	0.88	1.92
	2	1.23	0.12	**< 0.00005**	0.79	1.68
	3	0.93	0.11	**< 0.00005**	0.53	1.34
	4	0.78	0.10	**< 0.00005**	0.40	1.17
	5	0.61	0.09	**< 0.00005**	0.24	0.97
	6	0.25	0.07	0.2851	-0.04	0.53

8	1	1.46	0.13	**< 0.00005**	0.97	1.95
	2	1.29	0.11	**< 0.00005**	0.85	1.74
	3	0.99	0.10	**< 0.00005**	0.62	1.37
	4	0.84	0.09	**< 0.00005**	0.49	1.20
	5	0.67	0.09	**< 0.00005**	0.31	1.02
	6	0.31	0.08	0.1250	-0.02	0.63
	7	0.06	0.08	1.0	-0.25	0.37

9	1	1.54	0.15	**< 0.00005**	0.96	2.12
	2	1.37	0.13	**< 0.00005**	0.86	1.88
	3	1.07	0.11	**< 0.00005**	0.63	1.51
	4	0.92	0.11	**< 0.00005**	0.51	1.34
	5	0.75	0.10	**< 0.00005**	0.37	1.12
	6	0.39	0.08	**0.0012**	0.08	0.70
	7	0.14	0.09	1.0	-0.20	0.48
	8	0.08	0.07	1.0	-0.21	0.37

10	1	1.63	0.14	**< 0.00005**	1.07	2.18
	2	1.46	0.13	**< 0.00005**	0.96	1.96
	3	1.16	0.11	**< 0.00005**	0.74	1.58
	4	1.01	0.10	**< 0.00005**	0.62	1.41
	5	0.83	0.10	**< 0.00005**	0.47	1.20
	6	0.47	0.08	**< 0.00005**	0.15	0.80
	7	0.23	0.08	0.9955	-0.07	0.52
	8	0.17	0.07	1.0	-0.09	0.43
	9	0.09	0.06	1.0	-0.13	0.30

11	1	1.71	0.16	**< 0.00005**	1.08	2.33
	2	1.54	0.15	**< 0.00005**	0.96	2.13
	3	1.24	0.13	**< 0.00005**	0.75	1.73
	4	1.09	0.12	**< 0.00005**	0.61	1.58
	5	0.92	0.12	**< 0.00005**	0.47	1.36
	6	0.56	0.10	**< 0.00005**	0.17	0.94
	7	0.31	0.10	0.4559	-0.06	0.68
	8	0.25	0.09	1.0	-0.11	0.61
	9	0.17	0.09	1.0	-0.16	0.50
	10	0.08	0.07	1.0	-0.19	0.36

12	1	1.88	0.16	**< 0.00005**	1.28	2.48
	2	1.72	0.14	**< 0.00005**	1.16	2.27
	3	1.41	0.12	**< 0.00005**	0.94	1.89
	4	1.27	0.12	**< 0.00005**	0.81	1.72
	5	1.09	0.11	**< 0.00005**	0.65	1.52
	6	0.73	0.09	**< 0.00005**	0.39	1.07
	7	0.48	0.09	**0.0002**	0.12	0.84
	8	0.42	0.10	**0.0065**	0.05	0.79
	9	0.34	0.09	**0.0289**	0.01	0.67
	10	0.25	0.08	0.3524	-0.04	0.55
	11	0.17	0.07	1.0	-0.09	0.44

13	1	1.99	0.16	**< 0.00005**	1.38	2.59
	2	1.82	0.15	**< 0.00005**	1.26	2.38
	3	1.52	0.13	**< 0.00005**	1.03	2.01
	4	1.37	0.12	**< 0.00005**	0.90	1.84
	5	1.19	0.12	**< 0.00005**	0.75	1.64
	6	0.83	0.09	**< 0.00005**	0.47	1.20
	7	0.59	0.10	**< 0.00005**	0.22	0.95
	8	0.53	0.10	**0.0002**	0.14	0.91
	9	0.45	0.09	**0.0007**	0.10	0.80
	10	0.36	0.09	**0.0147**	0.03	0.69
	11	0.28	0.07	0.0684	-0.01	0.56
	12	0.11	0.06	1.0	-0.14	0.35

14	1	2.02	0.16	**< 0.00005**	1.39	2.64
	2	1.85	0.15	**< 0.00005**	1.27	2.43
	3	1.55	0.13	**< 0.00005**	1.04	2.06
	4	1.40	0.12	**< 0.00005**	0.93	1.87
	5	1.22	0.12	**< 0.00005**	0.77	1.68
	6	0.86	0.09	**< 0.00005**	0.50	1.23
	7	0.62	0.10	**< 0.00005**	0.25	0.99
	8	0.56	0.10	**0.0001**	0.16	0.96
	9	0.48	0.09	**0.0001**	0.13	0.83
	10	0.39	0.08	**0.0016**	0.07	0.71
	11	0.31	0.08	0.0689	-0.01	0.62
	12	0.14	0.06	1.0	-0.09	0.36
	13	0.03	0.06	1.0	-0.20	0.27

15	1	2.09	0.16	**< 0.00005**	1.46	2.72
	2	1.92	0.16	**< 0.00005**	1.32	2.53
	3	1.62	0.13	**< 0.00005**	1.10	2.14
	4	1.47	0.13	**< 0.00005**	0.97	1.98
	5	1.29	0.12	**< 0.00005**	0.81	1.77
	6	0.94	0.11	**< 0.00005**	0.51	1.36
	7	0.69	0.12	**< 0.00005**	0.24	1.14
	8	0.63	0.10	**< 0.00005**	0.24	1.02
	9	0.55	0.10	**< 0.00005**	0.17	0.93
	10	0.46	0.09	**0.0002**	0.12	0.80
	11	0.38	0.09	**0.0065**	0.05	0.71
	12	0.21	0.08	1.0	-0.12	0.53
	13	0.10	0.07	1.0	-0.17	0.38
	14	0.07	0.07	1.0	-0.21	0.35

16	1	2.21	0.17	**< 0.00005**	1.56	2.87
	2	2.05	0.16	**< 0.00005**	1.42	2.68
	3	1.75	0.14	**< 0.00005**	1.19	2.30
	4	1.60	0.13	**< 0.00005**	1.10	2.09
	5	1.42	0.14	**< 0.00005**	0.90	1.94
	6	1.06	0.12	**< 0.00005**	0.61	1.52
	7	0.81	0.12	**< 0.00005**	0.35	1.27
	8	0.76	0.12	**< 0.00005**	0.30	1.22
	9	0.68	0.11	**< 0.00005**	0.25	1.10
	10	0.59	0.10	**< 0.00005**	0.19	0.98
	11	0.51	0.10	**0.0003**	0.13	0.88
	12	0.33	0.09	**0.0420**	0.00	0.66
	13	0.23	0.09	1.0	-0.10	0.56
	14	0.20	0.08	1.0	-0.11	0.51
	15	0.13	0.07	1.0	-0.16	0.41

17	1	2.46	0.16	**< 0.00005**	1.84	3.07
	2	2.29	0.15	**< 0.00005**	1.72	2.86
	3	1.99	0.14	**< 0.00005**	1.46	2.53
	4	1.84	0.13	**< 0.00005**	1.35	2.33
	5	1.67	0.13	**< 0.00005**	1.17	2.16
	6	1.31	0.11	**< 0.00005**	0.86	1.75
	7	1.06	0.12	**< 0.00005**	0.59	1.53
	8	1.00	0.11	**< 0.00005**	0.56	1.44
	9	0.92	0.10	**< 0.00005**	0.52	1.32
	10	0.83	0.10	**< 0.00005**	0.43	1.23
	11	0.75	0.10	**< 0.00005**	0.36	1.14
	12	0.58	0.09	**< 0.00005**	0.22	0.94
	13	0.47	0.08	**< 0.00005**	0.17	0.77
	14	0.44	0.09	**0.0007**	0.10	0.79
	15	0.37	0.08	**0.0016**	0.07	0.67
	16	0.24	0.08	0.9765	-0.07	0.56

18	1	2.46	0.17	**< 0.00005**	1.79	3.13
	2	2.30	0.16	**< 0.00005**	1.66	2.93
	3	2.00	0.15	**< 0.00005**	1.42	2.57
	4	1.85	0.13	**< 0.00005**	1.33	2.37
	5	1.67	0.14	**< 0.00005**	1.12	2.22
	6	1.31	0.12	**< 0.00005**	0.84	1.78
	7	1.06	0.13	**< 0.00005**	0.58	1.55
	8	1.01	0.12	**< 0.00005**	0.54	1.47
	9	0.93	0.11	**< 0.00005**	0.51	1.34
	10	0.84	0.11	**< 0.00005**	0.42	1.25
	11	0.75	0.11	**< 0.00005**	0.33	1.18
	12	0.58	0.10	**< 0.00005**	0.19	0.98
	13	0.48	0.10	**0.0015**	0.09	0.86
	14	0.45	0.10	**0.0043**	0.06	0.83
	15	0.38	0.09	**0.0083**	0.04	0.71
	16	0.25	0.08	0.7876	-0.07	0.57

19	1	2.54	0.17	**< 0.00005**	1.87	3.20
	2	2.37	0.17	**< 0.00005**	1.72	3.02
	3	2.07	0.15	**< 0.00005**	1.48	2.66
	4	1.92	0.14	**< 0.00005**	1.36	2.47
	5	1.74	0.15	**< 0.00005**	1.18	2.31
	6	1.38	0.13	**< 0.00005**	0.88	1.88
	7	1.14	0.14	**< 0.00005**	0.60	1.67
	8	1.08	0.12	**< 0.00005**	0.60	1.55
	9	1.00	0.12	**< 0.00005**	0.54	1.45
	10	0.91	0.12	**< 0.00005**	0.46	1.35
	11	0.83	0.11	**< 0.00005**	0.40	1.25
	12	0.66	0.11	**< 0.00005**	0.21	1.10
	13	0.55	0.11	**0.0004**	0.13	0.97
	14	0.52	0.11	**0.0011**	0.10	0.93
	15	0.45	0.08	**0.0001**	0.12	0.77
	16	0.32	0.09	0.1837	-0.03	0.68
	17	0.08	0.08	1.0	-0.23	0.39
	18	0.07	0.07	1.0	-0.20	0.35

20	1	2.51	0.18	**< 0.00005**	1.82	3.20
	2	2.35	0.17	**< 0.00005**	1.68	3.01
	3	2.04	0.15	**< 0.00005**	1.45	2.64
	4	1.90	0.15	**< 0.00005**	1.32	2.47
	5	1.72	0.15	**< 0.00005**	1.12	2.31
	6	1.36	0.13	**< 0.00005**	0.85	1.87
	7	1.11	0.14	**< 0.00005**	0.57	1.65
	8	1.05	0.13	**< 0.00005**	0.54	1.56
	9	0.97	0.12	**< 0.00005**	0.51	1.43
	10	0.88	0.12	**< 0.00005**	0.41	1.35
	11	0.80	0.12	**< 0.00005**	0.34	1.26
	12	0.63	0.12	**0.0001**	0.18	1.08
	13	0.53	0.12	**0.0035**	0.08	0.97
	14	0.49	0.11	**0.0045**	0.07	0.92
	15	0.42	0.09	**0.0028**	0.07	0.78
	16	0.30	0.09	0.4107	-0.06	0.65
	17	0.05	0.10	1.0	-0.32	0.42
	18	0.05	0.07	1.0	-0.22	0.32
	19	-0.03	0.06	1.0	-0.25	0.19

21	1	2.56	0.18	**< 0.00005**	1.87	3.25
	2	2.40	0.17	**< 0.00005**	1.72	3.07
	3	2.10	0.16	**< 0.00005**	1.46	2.73
	4	1.95	0.15	**< 0.00005**	1.37	2.52
	5	1.77	0.15	**< 0.00005**	1.18	2.36
	6	1.41	0.14	**< 0.00005**	0.88	1.94
	7	1.16	0.15	**< 0.00005**	0.58	1.74
	8	1.10	0.14	**< 0.00005**	0.57	1.63
	9	1.02	0.14	**< 0.00005**	0.50	1.55
	10	0.93	0.13	**< 0.00005**	0.42	1.45
	11	0.85	0.13	**< 0.00005**	0.35	1.35
	12	0.68	0.13	**0.0002**	0.18	1.18
	13	0.58	0.12	**0.0010**	0.12	1.03
	14	0.54	0.12	**0.0028**	0.09	1.00
	15	0.47	0.10	**0.0021**	0.08	0.86
	16	0.35	0.11	0.6678	-0.09	0.78
	17	0.10	0.09	1.0	-0.26	0.47
	18	0.10	0.09	1.0	-0.25	0.44
	19	0.03	0.06	1.0	-0.21	0.26
	20	0.05	0.08	1.0	-0.25	0.36

22	1	2.53	0.18	**< 0.00005**	1.85	3.21
	2	2.37	0.18	**< 0.00005**	1.68	3.05
	3	2.07	0.17	**< 0.00005**	1.43	2.71
	4	1.92	0.16	**< 0.00005**	1.32	2.52
	5	1.74	0.16	**< 0.00005**	1.12	2.36
	6	1.38	0.15	**< 0.00005**	0.80	1.96
	7	1.13	0.15	**< 0.00005**	0.55	1.72
	8	1.07	0.14	**< 0.00005**	0.53	1.62
	9	0.99	0.14	**< 0.00005**	0.45	1.53
	10	0.91	0.13	**< 0.00005**	0.39	1.42
	11	0.82	0.14	**< 0.00005**	0.29	1.36
	12	0.65	0.13	**0.0009**	0.14	1.17
	13	0.55	0.13	**0.0128**	0.05	1.05
	14	0.52	0.13	**0.0289**	0.02	1.01
	15	0.45	0.11	**0.0167**	0.03	0.86
	16	0.32	0.11	1.0	-0.10	0.73
	17	0.07	0.11	1.0	-0.36	0.51
	18	0.07	0.11	1.0	-0.35	0.49
	19	0.00	0.09	1.0	-0.35	0.34
	20	0.02	0.09	1.0	-0.31	0.36
	21	-0.03	0.09	1.0	-0.36	0.31

23	1	2.68	0.19	**< 0.00005**	1.93	3.43
	2	2.52	0.19	**< 0.00005**	1.78	3.26
	3	2.22	0.18	**< 0.00005**	1.51	2.92
	4	2.07	0.17	**< 0.00005**	1.39	2.74
	5	1.89	0.18	**< 0.00005**	1.21	2.57
	6	1.53	0.16	**< 0.00005**	0.90	2.16
	7	1.28	0.17	**< 0.00005**	0.64	1.93
	8	1.23	0.17	**< 0.00005**	0.59	1.86
	9	1.15	0.16	**< 0.00005**	0.52	1.77
	10	1.06	0.15	**< 0.00005**	0.46	1.65
	11	0.97	0.15	**< 0.00005**	0.40	1.55
	12	0.80	0.15	**0.0001**	0.24	1.37
	13	0.70	0.14	**0.0005**	0.16	1.24
	14	0.67	0.14	**0.0021**	0.12	1.22
	15	0.60	0.12	**0.0011**	0.12	1.07
	16	0.47	0.12	0.0655	-0.01	0.95
	17	0.22	0.12	1.0	-0.23	0.68
	18	0.22	0.12	1.0	-0.24	0.68
	19	0.15	0.10	1.0	-0.25	0.54
	20	0.17	0.10	1.0	-0.22	0.57
	21	0.12	0.10	1.0	-0.27	0.51
	22	0.15	0.10	1.0	-0.23	0.53

24	1	2.73	0.20	**< 0.00005**	1.95	3.51
	2	2.57	0.20	**< 0.00005**	1.79	3.35
	3	2.27	0.19	**< 0.00005**	1.51	3.02
	4	2.12	0.18	**< 0.00005**	1.41	2.82
	5	1.94	0.19	**< 0.00005**	1.21	2.67
	6	1.58	0.18	**< 0.00005**	0.90	2.26
	7	1.33	0.18	**< 0.00005**	0.64	2.03
	8	1.27	0.18	**< 0.00005**	0.59	1.96
	9	1.19	0.17	**< 0.00005**	0.53	1.86
	10	1.11	0.17	**< 0.00005**	0.46	1.75
	11	1.02	0.17	**< 0.00005**	0.38	1.67
	12	0.85	0.17	**0.0003**	0.21	1.49
	13	0.75	0.16	**0.0024**	0.13	1.37
	14	0.72	0.16	**0.0062**	0.09	1.34
	15	0.64	0.14	**0.0041**	0.09	1.20
	16	0.52	0.14	0.0758	-0.02	1.05
	17	0.27	0.14	1.0	-0.25	0.80
	18	0.27	0.13	1.0	-0.25	0.79
	19	0.20	0.12	1.0	-0.27	0.67
	20	0.22	0.12	1.0	-0.22	0.67
	21	0.17	0.12	1.0	-0.30	0.64
	22	0.20	0.10	1.0	-0.19	0.58
	23	0.05	0.07	1.0	-0.21	0.31

SE: standard error; CI: confidence interval. Significant *P* values in bold.

**Table 4:** Descriptive statistics for the scores given by the three groups of raters to photographs with altered gonial heights. In images 1 to 9, the gonion was, respectively, 4.5 mm above the mouth corner, at the level of mouth corner, 4.5 mm below the mouth corner, 9 mm, 13.5 mm, 18 mm, 22.5 mm, 27 mm, and 31.5 mm below the mouth corner.

Image	Sex	OMFS					Orthodontists					Laypersons				
		*N*	Mean	SD	Min	Max	*N*	Mean	SD	Min	Max	*N*	Mean	SD	Min	Max
O1	Female	11	7.36	1.03	6	9	19	7.05	1.68	3	10	34	6.35	1.77	4	10
	Male	21	6.05	1.75	2	9	14	6.57	1.87	3	9	28	5.79	2.32	0	10
	Both	32	6.50	1.65	2	9	33	6.85	1.75	3	10	62	6.10	2.04	0	10

O2	Female	11	7.18	0.98	5	8	19	6.95	1.58	4	10	34	6.41	1.65	4	10
	Male	21	6.00	1.67	2	9	14	6.57	1.70	3	9	28	5.86	2.01	1	10
	Both	32	6.41	1.56	2	9	33	6.79	1.62	3	10	62	6.16	1.83	1	10

O3	Female	11	6.91	0.94	5	8	19	6.63	1.74	3	10	34	6.50	1.76	3	10
	Male	21	5.95	1.75	2	9	14	6.36	2.06	3	9	28	5.89	1.81	1	10
	Both	32	6.28	1.57	2	9	33	6.52	1.86	3	10	62	6.23	1.80	1	10

O4	Female	11	6.55	1.57	4	9	19	6.74	1.66	4	10	34	6.41	2.03	0	10
	Male	21	6.00	2.02	2	10	14	5.93	1.98	2	9	28	5.82	2.04	1	10
	Both	32	6.19	1.87	2	10	33	6.39	1.82	2	10	62	6.15	2.04	0	10

O5	Female	11	6.27	1.62	4	9	19	6.11	1.85	3	10	34	6.29	2.22	0	10
	Male	21	5.76	2.17	2	10	14	5.57	2.47	1	9	28	5.79	1.93	1	10
	Both	32	5.94	1.98	2	10	33	5.88	2.12	1	10	62	6.06	2.10	0	10

O6	Female	11	5.73	1.90	3	8	19	5.58	1.98	3	10	34	6.24	2.19	0	10
	Male	21	5.38	2.27	1	10	14	5.71	2.20	2	9	28	5.96	2.13	1	10
	Both	32	5.50	2.13	1	10	33	5.64	2.04	2	10	62	6.11	2.15	0	10

O7	Female	11	5.45	1.57	3	8	19	5.37	2.22	2	10	34	6.21	2.28	0	10
	Male	21	5.19	2.25	1	10	14	5.43	2.38	1	9	28	5.50	2.08	1	10
	Both	32	5.28	2.02	1	10	33	5.39	2.25	1	10	62	5.89	2.20	0	10

O8	Female	11	5.18	1.60	3	8	19	4.79	2.44	1	10	34	5.79	2.35	0	10
	Male	21	4.86	2.39	1	10	14	4.93	2.56	0	8	28	5.32	2.31	1	10
	Both	32	4.97	2.13	1	10	33	4.85	2.45	0	10	62	5.58	2.32	0	10

O9	Female	11	4.91	1.76	3	9	19	4.53	2.55	1	10	34	5.44	2.27	0	10
	Male	21	4.48	2.34	1	10	14	4.64	2.59	0	8	28	4.82	2.58	1	10
	Both	32	4.63	2.14	1	10	33	4.58	2.53	0	10	62	5.16	2.42	0	10

O: three-quarter oblique-view image; SD: standard deviation; Min: minimum; Max: maximum.

**Table 5:** The Bonferroni test results comparing the esthetic scores of the 9 Perceptometric images related to the gonial height. In images 1 to 9, the gonion was, respectively, 4.5 mm above the mouth corner, at the level of mouth corner, 4.5 mm below the mouth corner, 9 mm, 13.5 mm, 18 mm, 22.5 mm, 27 mm, and 31.5 mm below the mouth corner.

Image I	Image J	Difference (I-J)	SE	*P*	95% CI	
2	1	-0.03	0.06	1.0	-0.24	0.18

3	1	-0.16	0.09	1.0	-0.46	0.15
	2	-0.12	0.07	1.0	-0.36	0.11

4	1	-0.29	0.12	0.5380	-0.68	0.10
	2	-0.26	0.10	0.5096	-0.59	0.08
	3	-0.14	0.08	1.0	-0.38	0.11

5	1	-0.57	0.14	**0.0042**	-1.03	-0.10
	2	-0.53	0.13	**0.0027**	-0.96	-0.11
	3	-0.41	0.10	**0.0013**	-0.73	-0.10
	4	-0.28	0.07	**0.0084**	-0.52	-0.04

6	1	-0.77	0.14	**< 0.00005**	-1.21	-0.32
	2	-0.73	0.13	**< 0.00005**	-1.14	-0.32
	3	-0.61	0.10	**< 0.00005**	-0.94	-0.28
	4	-0.48	0.08	**< 0.00005**	-0.72	-0.23
	5	-0.20	0.08	0.4606	-0.46	0.06

7	1	-1.02	0.15	**< 0.00005**	-1.50	-0.53
	2	-0.98	0.14	**< 0.00005**	-1.44	-0.53
	3	-0.86	0.11	**< 0.00005**	-1.22	-0.50
	4	-0.73	0.10	**< 0.00005**	-1.05	-0.40
	5	-0.45	0.09	**0.0001**	-0.75	-0.15
	6	-0.25	0.08	0.0573	-0.50	0.00

8	1	-1.40	0.17	**< 0.00005**	-1.94	-0.86
	2	-1.37	0.16	**< 0.00005**	-1.88	-0.85
	3	-1.24	0.14	**< 0.00005**	-1.69	-0.80
	4	-1.11	0.12	**< 0.00005**	-1.51	-0.70
	5	-0.83	0.11	**< 0.00005**	-1.20	-0.46
	6	-0.63	0.10	**< 0.00005**	-0.95	-0.31
	7	-0.38	0.08	**0.0003**	-0.65	-0.12

9	1	-1.74	0.19	**< 0.00005**	-2.37	-1.11
	2	-1.71	0.18	**< 0.00005**	-2.31	-1.11
	3	-1.59	0.16	**< 0.00005**	-2.10	-1.07
	4	-1.45	0.15	**< 0.00005**	-1.95	-0.95
	5	-1.18	0.14	**< 0.00005**	-1.62	-0.73
	6	-0.97	0.14	**< 0.00005**	-1.43	-0.52
	7	-0.73	0.12	**< 0.00005**	-1.12	-0.33
	8	-0.34	0.10	**0.0156**	-0.65	-0.03

SE: standard error; CI: confidence interval. Significant *P* values in bold.

## Data Availability

The data are available from the authors upon reasonable request.
